# Translational Identification of Transcriptional Signatures of Major Depression and Antidepressant Response

**DOI:** 10.3389/fnmol.2017.00248

**Published:** 2017-08-08

**Authors:** Mylène Hervé, Aurélie Bergon, Anne-Marie Le Guisquet, Samuel Leman, Julia-Lou Consoloni, Nicolas Fernandez-Nunez, Marie-Noëlle Lefebvre, Wissam El-Hage, Raoul Belzeaux, Catherine Belzung, El Chérif Ibrahim

**Affiliations:** ^1^Aix Marseille Univ, CNRS, CRN2M UMR 7286 Marseille, France; ^2^FondaMental, Fondation de Recherche et de Soins en Santé Mentale Créteil, France; ^3^Aix Marseille Univ, INSERM, TAGC UMR_S 1090 Marseille, France; ^4^INSERM U930 Eq 4, UFR Sciences et Techniques, Université François Rabelais Tours, France; ^5^AP-HM, Hôpital Sainte Marguerite, Pôle de Psychiatrie Universitaire Solaris Marseille, France; ^6^TGML, Platform IbiSA, Aix Marseille Univ, INSERM U1090, TAGC Marseille, France; ^7^CIC-CPCET, AP-HM, Hôpital La Timone Marseille, France; ^8^CHRU de Tours, Clinique Psychiatrique Universitaire Tours, France; ^9^INSERM CIC 1415, Centre d’Investigation Clinique, CHRU de Tours Tours, France; ^10^McGill Group for Suicide Studies, Douglas Mental Health University Institute, Department of Psychiatry, McGill University Montreal, QC, Canada; ^11^Aix Marseille Univ, CNRS, INT, Inst Neurosci Timone UMR 7289 Marseille, France

**Keywords:** chronic stress, depression, mRNA, blood, transcriptomics, biomarker, antidepressant

## Abstract

Major depressive disorder (MDD) is a highly prevalent mental illness whose therapy management remains uncertain, with more than 20% of patients who do not achieve response to antidepressants. Therefore, identification of reliable biomarkers to predict response to treatment will greatly improve MDD patient medical care. Due to the inaccessibility and lack of brain tissues from living MDD patients to study depression, researches using animal models have been useful in improving sensitivity and specificity of identifying biomarkers. In the current study, we used the unpredictable chronic mild stress (UCMS) model and correlated stress-induced depressive-like behavior (*n* = 8 unstressed vs. 8 stressed mice) as well as the fluoxetine-induced recovery (*n* = 8 stressed and fluoxetine-treated mice vs. 8 unstressed and fluoxetine-treated mice) with transcriptional signatures obtained by genome-wide microarray profiling from whole blood, dentate gyrus (DG), and the anterior cingulate cortex (ACC). Hierarchical clustering and rank-rank hypergeometric overlap (RRHO) procedures allowed us to identify gene transcripts with variations that correlate with behavioral profiles. As a translational validation, some of those transcripts were assayed by RT-qPCR with blood samples from 10 severe major depressive episode (MDE) patients and 10 healthy controls over the course of 30 weeks and four visits. Repeated-measures ANOVAs revealed candidate trait biomarkers (*ARHGEF1*, *CMAS*, *IGHMBP2*, *PABPN1* and *TBC1D10C*), whereas univariate linear regression analyses uncovered candidates state biomarkers (*CENPO*, *FUS* and *NUBP1*), as well as prediction biomarkers predictive of antidepressant response (*CENPO*, *NUBP1*). These data suggest that such a translational approach may offer new leads for clinically valid panels of biomarkers for MDD.

## Introduction

Major depressive disorder (MDD) is a highly prevalent mental illness characterized by altered emotional, cognitive and behavioral functions. Pathophysiology of depression is complex and is hypothesized to involve several biological processes, including neurotransmitter dysfunction, neuronal networks alteration, inadequate neuroendocrine stress response, unsynchronized circadian rhythms and chronic inflammation (Moylan et al., 2013). Major depression is therefore unlikely to have a single cause. Diagnosis of MDD still mainly relies on subjective evaluation, such as self-reporting of symptoms and clinical interviews (Young et al., 2016). Although modern pharmacological medications such as selective serotonin reuptake inhibitors (SSRIs) and more recently serotonin-norepinephrine reuptakes inhibitors (SNRIs, e.g., duloxetine), has demonstrated efficacy and potential to prevent negative consequences (such as suicidal behavior) associated with MDD (Girardi et al., 2009), the optimal therapies to manage MDD remain unclear, as more than 20% of MDD patients remain resistant to treatments and around 50% of the episodes are recurrent (Kessler, 2003; Möller, 2008). Therefore, there is a need for improvement of MDD patient medical care and identification of reliable biomarkers could help in diagnosis, classification of MDD subtypes, and monitoring of disease progression (Jentsch et al., 2015; Young et al., 2016).

The term biomarker can be understood in different ways. A biomarker can provide information about the pathophysiology of a disease, as well as be used as an objective tool to validate a diagnosis. It can also predict disease progression or provide prediction on treatment response (McMahon, 2014). Classically, the most common biomarker concepts correspond to individual features used as indicators of a disease state (diagnostic biomarker), or even predictor of clinical outcome following the treatment (treatment biomarker, Papakostas and Fava, 2008; Davis et al., 2015). Generally considered as a biological variable, a biomarker is not limited to this definition and can encompass functional tests as fMRI or surveys (Leuchter et al., 2010; Phillips et al., 2015).

Despite decades of research in the depression field, no evidence of depression-related biomarkers have been identified (Gururajan et al., 2016). Although some potential biomarkers based on biological theories of depression etiology have emerged, none are being used in clinical practice (Breitenstein et al., 2014). Difficulty in identifying candidate depression biomarkers is due to limited knowledge of depression’s etiology and the absence of a phenotype attributed to a single gene in depression pathophysiology. This is further reinforced by the difficulty in studying brain dysfunctions solely by relying on peripheral biological variations. Indeed, inaccessibility to brain tissues from living MDD patients constrained research to using blood and occasionally cerebrospinal fluid to study genetic alterations in depressive subjects (Wan et al., 2015; Hestad et al., 2016), making it almost impossible to draw any direct correlations between changes observed in the brain. To overcome this issue and to extend our understanding of biological pathways mediating individual differences in behavior and risk for psychopathology of major depression, translational research has been established using animal models of depression (Joeyen-Waldorf et al., 2012; Pajer et al., 2012; Issler et al., 2014; Arloth et al., 2015; Qesseveur et al., 2016). Using this method, it is possible to test animal tissues from both central and peripheral locations, identify candidate biomarkers, and assess correlation of these marker variations of expression in accessible human MDD samples with etiological hypotheses of depression. Translational studies have the advantage of improving the sensitivity and specificity of biomarker researches (Bertsch et al., 2005). Notably, most biomarkers identification efforts for neuropsychiatric disorders have relied on transcriptomics rather than proteomics (Breen et al., 2016), the former being less expensive while yielding more exhaustive biological data than the latter.

With the goal of identifying potential diagnostic or predictive biomarkers of depression, we undertook a translational approach that correlates data from a rodent model of depression with data from human blood samples. We utilized the unpredictable chronic mild stress (UCMS) model, which has contributed to the elucidation of the pathophysiological mechanisms of depression such as decreased neurogenesis, hypothalamic-pituitary-adrenal (HPA) axis alterations and maladaptive changes in amygdala (Surget et al., 2008; Sibille et al., 2009; Nollet et al., 2013). We aimed to correlate depressive-like behavior induced by UCMS protocol and fluoxetine-induced recovery with transcriptional signatures from whole blood as well as the hippocampus and the anterior cingulate cortex (ACC), two brain regions involved in mood regulation in which dysfunction has been reported in MDD (Rive et al., 2013; Jaworska et al., 2015; Wise et al., 2016). Since the hippocampus is known to have large variations in gene expression, we focused on the dentate gyrus (DG) where adult neurogenesis has been described and linked to psychiatric illness (Kohen et al., 2014). Hierarchical clustering procedures and classical statistical threshold methods allowed us to identify potential biomarkers with variations that correlate with behavioral profile. Moreover, we uncovered statistically significant overlaps between gene expression signatures from peripheral and central tissues by applying the innovative rank-rank hypergeometric overlap (RRHO) procedure. Finally, we validated our newly identified gene candidates with blood samples from a longitudinal human cohort of severe major depressive episode (MDE) patients. These patients were free of antidepressant at baseline, and monitored over 30 weeks for disease progression.

## Materials and Methods

### Animals

Thirty-two 8-week-old male BALB/c (Centre d’Elevage Janvier, Le Genest St. Isle, France) were divided into four groups. The first group (S-C, *Stressed-Control, n* = 8) of mice was subjected to UCMS procedure for 8 weeks (Nollet et al., 2013). The second group (NS-C, *No Stressed-Control*, *n* = 8) of mice was kept in standard housing conditions for 8 weeks as controls. In the third group (S-FLX, *Stressed-Fluoxetine*, *n* = 8), mice were subjected to UCMS procedure for 8 weeks and treated in parallel by fluoxetine during the last 6 weeks. The fourth group (NS-FLX, *No Stressed-Fluoxetine*, *n* = 8) of mice was unstressed but treated with fluoxetine during the last 6 weeks. Fluoxetine hydrochloride (Sequoia Research Products, Pangbourne, United Kingdom) was placed in drinking water at 120 mg/L and each mouse consumed 10–20 mg/kg/day of antidepressant treatment depending on the amount of water consumption. A diagram outlining the experimental protocol is presented in Supplementary Figure S1. At the end of the protocol, 0.5 mL of blood was collected from the submandibular vein and stabilized with 1.3 mL RNAlater^®^ solution (Life Technologies, Ambion, Austin, TX, USA). Mice were euthanized by CO_2_ inhalation. Brains were rapidly extracted and microdissected to recover ACC and DG samples.

All experiments on mice were carried out according to policies on the care and use of laboratory animals of European Community legislation 2010/63/EU. The local Ethics Committee (CEEAVdL-19) approved the protocols used in this study (protocol number 2011-06-10).

### UCMS

Mice from the NS-C and NS-FLX groups were housed in groups of four in standard cages, whereas the UCMS-exposed mice were isolated in individual home cages with no physical contact with other mice. The stressors used were varied and applied in a different sequence each week in order to avoid habituation (see Supplementary Materials and Methods).

### Mice Behavior

Weight and coat state were measured weekly, as markers of UCMS-induced depressive-like behavior, except for the last week before sacrifice, when coat state from seven different areas of the body was recorded twice, separated by 3-day intervals (see Supplementary Materials and Methods, Supplementary Figure S1). At the end of the 8th week, a complementary test of nest building was performed just before sacrifice. The test was administered by isolating mice in their home cages (see Supplementary Materials and Methods). To assign stress susceptibility/resiliency and then extrapolate antidepressant response/nonresponse for each mouse, cutoffs were defined according to the distribution of the sum of both coat state measurements in NS-C and NS-FLX vs. S-C groups. Next, S-FLX mice were further divided into responders, S-FLX-R (sum of coat scores ≤2), and nonresponders, S-FLX-NR (sum of coat scores >2).

### Human Cohort

Ten MDE patients were selected from a larger French cohort (see Supplementary Materials and Methods). Patients were required to be free of antidepressants at baseline, and were matched for age and sex with 10 healthy controls enrolled in the south of France (Marseille) from the same cohort. Clinical assessments of both patients and controls were made at baseline (week 0), and at 2, 8 and 30 weeks after inclusion (Supplementary Table S1). All patients met the Diagnostic and Statistical Manual of mental disorders, fourth edition, Text Revision (DSM-IV-TR) criteria for MDE (American Psychiatric Association, 2000), presenting at least severe MDE (17-item Hamilton Depression Rating Scale, HDRS, score ≥20) (American Psychiatric Association, 2008). Venous blood (8–9 mL) was drawn from fasting MDE patients and healthy controls in EDTA tubes (Greiner Bio-One GmbH, Kremsmünster, Austria) and processed within 40 min at inclusion (V1), 2 (V2), 8 (V3) and 30 (V4) weeks after inclusion. Blood was passed through a LeukoLOCK™ filter (Life Technologies, Ambion), to capture total leukocyte population while eliminating red blood cells, platelets, and plasma. After rinsing with phosphate-buffered saline, the filter was flushed with a RNA*later*^®^ solution to stabilize the RNA in the captured leukocytes. The filter was then stored at −80°C before processing.

All experiments on human subjects were conducted in accordance to the latest version of the Declaration of Helsinki. The project was approved by the local ethics committee (CPP Sud Méditerranée II, Marseille, France, study registered under number 2011-A00661-40).

### RNA Isolation

For mice samples, total RNAs were purified from the blood using the Mouse RiboPure-Blood RNA isolation kit (Life Technologies, Ambion), according to manufacturer’s recommendations, and from brain samples using the mirVana miRNA isolation kit (Life Technologies, Ambion) after mechanical grinding of the tissues. For human samples, leukocytes trapped on LeukoLOCK filters were lysed with TRI reagent (Ambion) and mixed with Bromo-3-chloro-propane (Sigma-Aldrich, St. Louis, MO, USA). After centrifugation, total RNA from the aqueous phase was precipitated with ethanol and then purified on spin cartridge. After washings, total RNAs were eluted with 0.1 mM EDTA. Both human and mice total RNA samples were subsequently submitted to DNase treatment (DNA-free^TM^ kit, Life Technologies, Ambion, Austin, TX, USA). RNA concentration was determined using a nanodrop ND-1000 spectrophotometer (Thermo Scientific, Waltham, MA, USA). RNA integrity was assessed on an Agilent 2100 Bioanalyzer (Agilent Technologies, Santa Clara, CA, USA).

### Microarray Assay

Sample amplification, labeling and hybridization onto Agilent whole mouse genome oligo microarrays (SurePrint G3 Mouse Gene Expression 8x60K Microarray v1, Agilent Technologies, Santa Clara, CA, USA) followed the one-color microarray-based gene expression analysis (Low Input Quick Amp Labeling) recommended by Agilent Technologies (see Supplementary Materials and Methods). The scanned images were analyzed with Agilent feature extraction software 10.5.1.1 using default parameters (protocol GE1_105_Dec08 and Grid 028004_D_F_20110722) to obtain background subtracted and spatially detrended processed signal intensities. Blood and brain data were independently normalized by quantile normalization using limma R/bioconductor package (v.2.16.4). All the procedures from control quality steps to normalized expression matrix data export, including normalization have been performed under R language with Limma R/Bioconductor library. The microarray data are available from the gene expression omnibus (GEO)^1^ under the series accession number GSE84185.

### Individual Assays for mRNA Expression Quantification

One microgram of total RNA was reverse transcribed using the High-Capacity cDNA Reverse Transcription kit (Life Technologies, Applied Biosystems, Foster City, CA, USA). Real-time PCR reactions were performed in duplicates using the TaqMan Universal PCR Master Mix II with no UNG (Life Technologies, Applied Biosystems) on 50 ng of the resulting cDNA, with an ABI PRISM 7900HT thermocycler under the following conditions: 10 min at 95°C, 50 cycles of 15 s at 95°C and 1 min at 60°C. Primers/TaqMan probe assays purchased from Applied Biosystems were used to determine the level of expression of the mouse and human candidate genes (Supplementary Table S2). A search into the microarray data for probes showing stable expression in the stressed, non-stressed and Flx-treated animals but also blood, DG and ACC, revealed *Rab5a* as a moderately expressed reference gene. For human expression, we used *CRYL1* as a reference gene for highly/moderately expressed genes and *SV2A* for weakly expressed genes (Belzeaux et al., 2012; Supplementary Table S2). Raw Ct values were obtained with manual baseline settings on the RQ Manager software (Applied Biosystems), and then the relative expression level of each mRNA was quantified by using the 2^−ΔΔCt^ method (Livak and Schmittgen, 2001).

### Statistical Analysis

#### Microarray Data

Fold Changes (FC) and parametric Student’s *t*-tests were generated under R. Expression matrix from either blood or brain samples were analyzed using the MultiExperiment Viewer 4 (MeV4, ref version MeV_4_8_1) to generate hierarchical clustering and Significance Analysis for Microarrays (SAM) set at FDR threshold <1%. RRHO test was applied to compare patterns of gene regulation between blood and brain regions under stress application (S-C vs. NS-C) and after fluoxetine treatment (S-FLX-R vs. S-C). RRHO identifies overlap between expression profiles in a threshold free manner to assess the degree and significance of overlap (Plaisier et al., 2010). Tests looking for over- and under-enrichment were used. Full differential expression lists were ranked by the −log10 (*P*-value) extracted from the *t* tests multiplied by the sign of the FC. The RRHO test was used to evaluate the overlap of differential expression lists between either blood and ACC, or blood and DG, or ACC and DG. A two-sided version of the test only looking for over-enrichment was used.

#### Behavior and Candidate Gene Validation

Statistical analyses on behavior and molecular validation data were assessed using the IBM SPSS Statistics v20 software, with threshold *P*-value set at 0.05. After ensuring the normal distribution of data with Shapiro-Wilk test, parametric Student’s *t*-test was used for weight gain and individual gene validation on mice samples. Non-parametric Mann-Whitney test was applied for nest building scores and coat scores. Transcriptional trajectories across a 30-week follow-up were compared between human patients and healthy controls by repeated-measures ANOVAs, which allowed the comparison of candidate gene mRNAs between groups, visits and the group by visit interaction. Linear regression analyses were conducted to determine if variations of candidate gene expression in MDE patients were associated to variations of clinical score assessed with the HDRS.

### Ontological Analysis

Gene lists were uploaded on DAVID (database for annotation, visualization and integrated discovery) Bioinformatics Ressources 6.7^2^ for identifying statistically relevant biological processes (Huang Da et al., 2009), with medium classification stringency and corrected *P*-value (Bonferroni) < 0.05.

## Results

### Depressive-Like Behavior Is Induced by Chronic Stress and Treated by Fluoxetine

**Figure 1:**
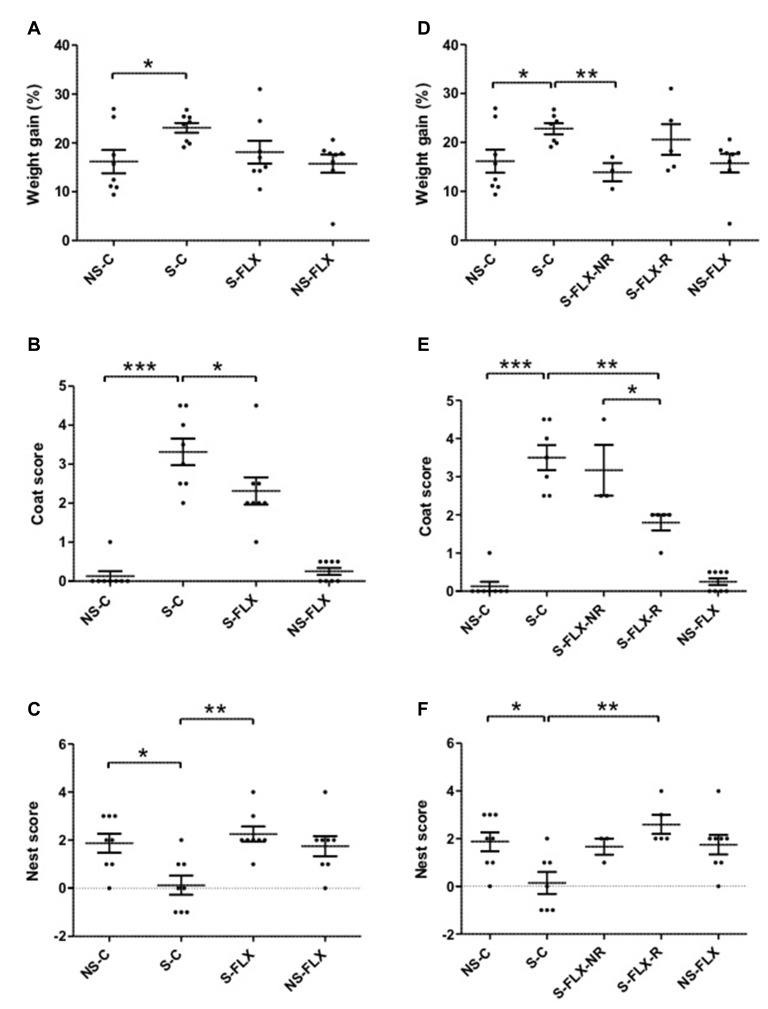
Behavioral tests assessing depressive-like parameters. After 8 weeks of unpredictable chronic mild stress (UCMS) protocol, mice were submitted to several behavioral tests evaluating depressive-like phenotypes. Results are represented as dot plots for the four original mice groups (left panel) or after reclassification according to their responder/nonresponder phenotype (right panel). **(A,D)** Individual weight variation calculated after UCMS; **(B,E)** addition of individual coat scores obtained 3 days before sacrifice to the one at the time of sacrifice; **(C–F)** individual difference between nest building scores obtained after 5 h and 24 h tests. Horizontal bars denote mean values and error bars denote standard error (*P < 0.05, **P < 0.01, ***P < 0.001 using parametric *t*-test **(A,D)** or non-parametric Mann-Whitney test **(B,C,E,F)**). NS, non-stressed; S, stressed; C, vehicule-treated; FLX, fluoxetine-treated; NR, nonresponder; R, responder.

To model appearance of depressive symptoms in MDE patients and recovery after antidepressant treatment, 16 mice were treated with UCMS protocol for 8 weeks and a second group of 16 mice were concomitantly kept in stress-free conditions. Half of each of the stressed and unstressed mice group (eight stressed and eight unstressed mice) was treated with fluoxetine during the last 6 weeks. The effects of chronic stress on susceptibility/resilience and response/nonresponse to antidepressant treatment have been evaluated by several behavioral tests. First, weight variation, one of the diagnostic criteria of depression, was measured each week. As represented in Figure 1A, all mice had gained about 15–25% of their starting weight. At the end of the UCMS protocol, stressed mice exhibited a significant increase of their weight compared to unstressed mice, which is counteracted by fluoxetine treatment (Figure 1A). In parallel, depressive-like physical alteration, assessed by the coat state score, which is a measurement of self-care, clearly distinguished unstressed mice (null or low, ≤1, score) from mice exposed to chronic stress (scores~3). Such depressive behavior was partly reversed by fluoxetine treatment (Figure 1B). Furthermore, evaluation of coat state score confirmed that stress protocol was mild in intensity as maximal scores along the 8-week monitoring never exceeded 2.5 in stressed mice (Supplementary Figure S2A). Loss of motivation/apathetic behavior was evaluated with the nest-building test a week before sacrifice. As observed in Figure 1C, stressed mice exhibited an important reduction of nest building score compared to unstressed mice, and fluoxetine treatment restored normal activity. Taken together, these results confirmed depressive-like behaviors induced by chronic mild stress in mice and its reversibility with chronic fluoxetine treatment.

### Blood Transcriptional Profiles Reflect Behavioral Variations within Groups of Mice

To identify potential biomarkers of a depressive-like state in mice, we conducted a genome-wide transcriptome analysis on all RNA samples extracted from blood and the DG and ACC regions of the brain, both of which play major roles in emotion processing and are known to be altered in affective disorders (Jaworska et al., 2015). Processing of the raw data resulted in 17,368 analyzable probes for blood and 33,264 probes for each brain region. Interestingly, we observed that the mean probe expression level was 2–3 fold higher in brain compared to blood samples (Supplementary Figure S3). Therefore, we separately analyzed blood and brain data to overcome any information that may be lost during normalization procedures of microarray data.

**Figure 2:**
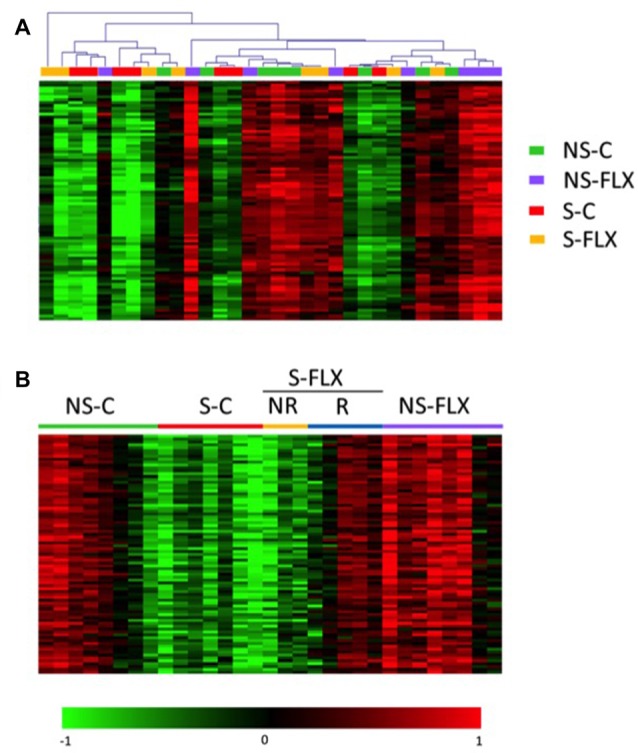
Heatmap representation of gene expression level in blood. Heatmap of 83 gene probes whose blood signature discriminate groups of mice. Overexpressed probes are in red and underexpressed in green. Normalized signal intensities were treated using the MultiExperiment Viewer (MeV) software by applying significance analysis for microarrays (SAM) test in unsupervised mode **(A)** or supervised mode based on the five reclassified groups **(B)**.

First, we tested the concordance between transcriptional data and the procedures applied to each mouse by performing an unsupervised partitioning of tissue microarray data using the MeV software. Figure 2A shows that, globally, each group of mice possesses a specific molecular signature in the blood that makes it different from other groups. This indicates that a blood molecular profiling can identify depressive-like phenotypes. The same evaluation conducted with brain signatures provided less clear-cut distinction between mice groups, especially for DG (Supplementary Figures S4A,B). Of note, some mice displayed a very different transcriptional pattern compared to other mice of the same group (Figure 2A, Supplementary Figures S4A,B). In addition, an internal dichotomous profile seemed to emerge within the stress and fluoxetine-treated mice that may be related to the different susceptible/resilient and responder/nonresponder phenotypes. Thus, the S-FLX mice were separated into two subcategories, as either responder (S-FLX-R, *n* = 5) or nonresponder (S-FLX-NR, *n* = 3) based on positive (coat score ≤2) or negative (coat score >2) response to fluoxetine treatment, respectively (Supplementary Table S3). In addition, one mouse, with a very distinct transcriptional pattern was removed from the S-C (*n* = 7) group. With this new classification, behavioral test results had a good correlation with scores and fluoxetine response (Figures 1D–F). Moreover, when examining how coat score evolved during the 6 weeks of antidepressant treatment in stressed mice, it appeared that responder mice demonstrated weaker maximal coat score than nonresponder animals (Supplementary Figure S2B), suggesting that the responder mice are more resilient to stress. Finally, to reflect a better molecular homogeneity within mice groups, we integrated our new mice classification into a supervised partitioning of transcriptional signatures using the same probes we used for the unsupervized clustering (Figure 2B, Supplementary Figures S4C,D).

### Identification of Potential Biomarkers using Threshold Method Analysis

**Figure 3:**
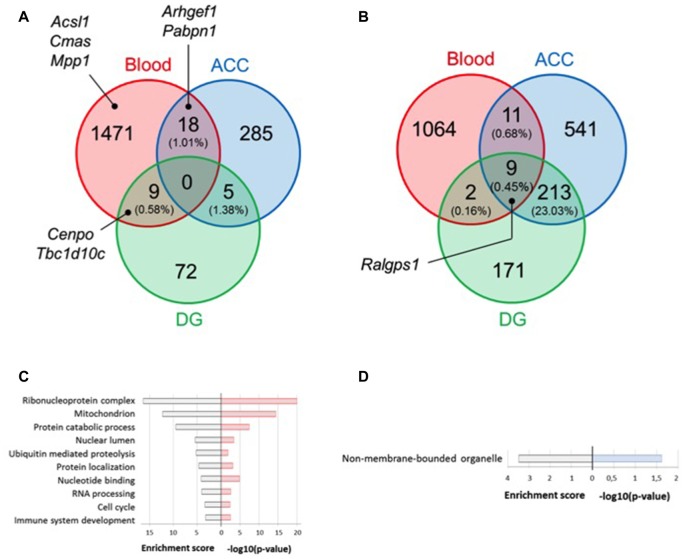
Selection of candidate genes according to convergent fold changes (FC) dysregulation between tissues. Venn diagrams represent overlap of significant dysregulated genes in blood and brain tissues under stress and fluoxetine (*P* < 0.05) treatment **(A)** or under stress condition only (*P* < 0.01 and FC > 1.2) **(B)**. Enriched gene ontology terms from the lists of dysregulated genes under stress and fluoxetine treatment (*P* < 0.05) in blood **(C)** and anterior cingulate cortex (ACC) **(D)**. For each scheme, enrichment score is indicated on the left side and −log10(P-value) on the right side.

To define state or trait biomarkers, for each tissue, we selected genes whose expression levels are modulated (*P* < 0.05) under stress (i.e., S-C vs. NS-C mice groups), and reversed by fluoxetine treatment (i.e., S-FLX-R vs. S-C mice groups). We also excluded genes whose expression varied under fluoxetine treatment without stress. Using this approach, no gene was shared by the three tissues, and only a poor overlap (<2%) existed between two tissues (Figure 3A and Supplementary Table S4A). Similarly, when the same lists were analyzed for gene ontology enrichment, no overlap could be found between all three tissues. Whereas DG could not reveal any significantly enriched biological process, only one was found for ACC relating to factors involved in gene expression: “non-membrane-bounded organelle” (Figure 3D). Biological processes enriched in blood include the ontological categories “ribonucleoprotein complex”, “nucleotide binding”, and “RNA processing”, in addition to processes frequently associated to depression such as “immune system development”, “ubiquitin-mediated proteolysis” and “mitochondrion” (Figure 3C).

From the dysregulated in both blood and ACC (18 genes), and blood and DG (nine genes), we found five and three genes (Supplementary Table S4A, underlined genes), respectively, that were also dysregulated in a previous transcriptome analysis we conducted from blood samples from MDE patients and healthy controls (Belzeaux et al., 2012). We selected among them, TBC1 domain family member 10C (*Tbcad10c*) and Centromere protein O (*Cenpo*) genes (for convergence between blood and DG) as well as the Rho guanine nucleotide exchange factor 1 (*Arhgef1*) and Poly(A) binding protein 1 (*Pabpn1*) genes (for convergence between blood and ACC) as candidate biomarkers (Figure 3A and Supplementary Table S4A, bold genes). Since our main goal was to identify blood biomarkers for clinical use, we further focused on blood data (the 1498 dysregulated genes, Figure 3A) and retained only the 70 ones with FDR <1% by SAM analysis of (NS-FLX + S-FLX-R) vs. (S-C + S-FLX-NR) groups of mice (Supplementary Table S5). Among these genes, 22 were also dysregulated in our previous transcriptome analysis on human blood samples (Belzeaux et al., 2012; Supplementary Table S5, underlines genes). Using this method, we selected the Acyl-CoA synthetase long-chain family member 1 (*Acsl1*), Cytidine monophosphate N-acetylneuraminic acid synthetase (*Cmas*), and Membrane palmitoylated protein 1 (*Mpp1*) as candidate genes underexpressed after stress and restored to basal level after fluoxetine treatment in responder mice (Figure 3A and Supplementary Table S5, bold genes). In addition, with the goal of finding blood biomarkers reflecting brain alterations, we also refined the selection parameters by focusing on genes specifically dysregulated by stress conditions by setting *P*-value threshold at 0.01 and FC >1.2 (Figure 3B and Supplementary Table S4B). We uncovered nine common dysregulated genes and we selected Ral GEF with PH domain and SH3 binding motif 1 (*Ralgps1*) as a potential candidate biomarker (Figure 3B and Supplementary Table S4B, bold genes).

**Figure 4:**
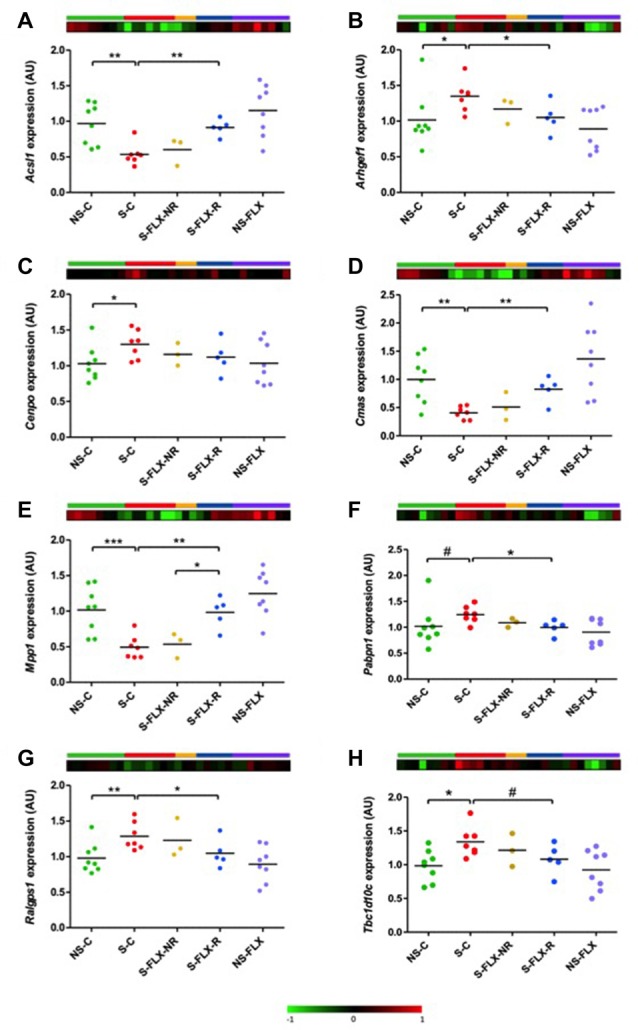
Validation of candidate gene expression in mice blood. Dot plots represent mean of expression of *Acsl1* **(A)**, *Arhgef1* **(B)**, *Cenpo* **(C)**, *Cmas* **(D)**, *Mpp1* **(E)**, *Pabpn1* **(F)**, *Ralgps1* **(G)** and *Tbc1d10c* **(H)** on mice blood samples analyzed by individual RT-qPCR. Mean of No Stressed-Control (NS-C) samples was used as a calibrator and *Rab5a* as a reference gene. Statistical analysis was realized using parametric *t*-test (^#^*P* < 0.1, *P < 0.05, **P < 0.01, ***P < 0.001). Heatmaps represent expression variation of the most significant microarray probe for each candidate gene.

Next, to validate expression dysregulations of these eight candidate genes, we performed individual RT-qPCR experiments on our mice samples. Results were normalized by *Rab5a*, a reference gene that was selected due to its stability in all groups of mice according to our transcriptome data. For all the eight tested candidate genes, we confirmed a stress-induced dysregulation reversed in responder mice by fluoxetine in blood tissue (Figure 4). Interestingly, these variations in blood (Figure 4) appeared to match behavioral observations (Figure 1), supporting the fact that a peripheral molecular signature is able to reflect a behavioral phenotype.

### Identification of New Potential Biomarkers using RRHO Method

**Figure 5:**
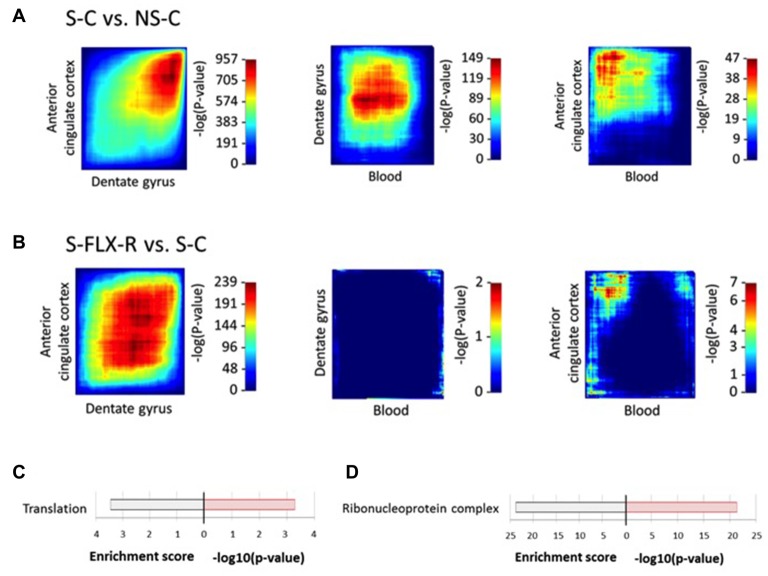
Inter-tissue differential expression pattern of transcriptional signatures under stress or fluoxetine treatment. Rank-rank hypergeometric overlap (RRHO) maps compare threshold-free differential expression between pairs of tissues (ACC-DG, left panel; blood-DG, middle panel; blood-ACC, right panel) after stress protocol **(A)** or with fluoxetine treatment **(B)**. Significance overlap was assessed by a hypergeometric test, color-coded. Enriched gene ontology terms from lists of the most co-dysregulated genes in NS-C vs. S-C (Supplementary Table S6B) in blood and dentate gyrus (DG) **(C)** and blood and ACC **(D)**. For each scheme, enrichment score is indicated on the left side and −log10(P-value) on the right side.

**Figure 6:**
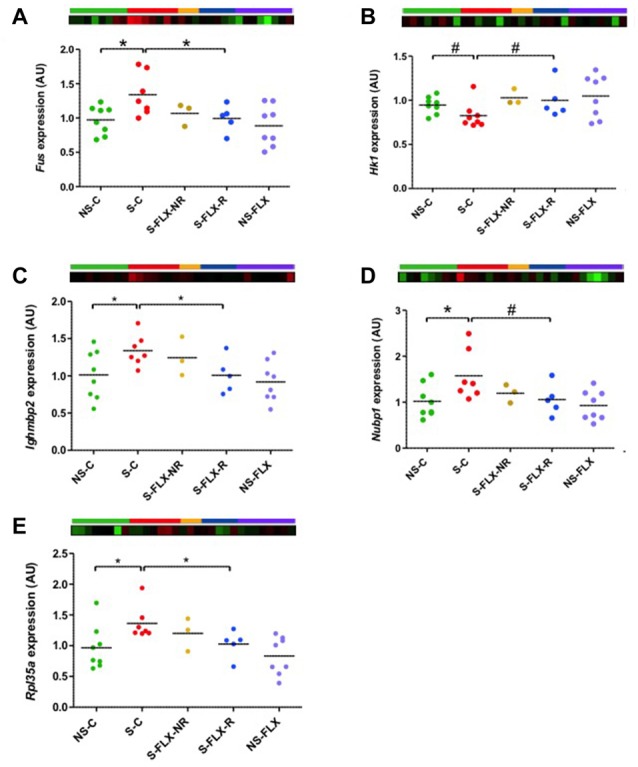
Validation of RRHO candidate gene expression in mice blood. Dot plots represent mean of expression of *Fus* **(A)**, *Hk1* **(B)**, *Ighmbp2* **(C)**, *Nubp1* **(D)** and *Rpl35a* **(E)** in mice blood samples analyzed by individual RT-qPCR. Mean of NS-C samples was used as a calibrator and *Rab5a* as a reference gene. Statistical analysis was realized using parametric *t*-tests (^#^*P* < 0.1, *P < 0.05). Heatmap represent expression variation of the most significant probe for each gene from the microarray data.

Previous identification of biomarkers employed classical techniques to identify significantly dysregulated genes by setting a differential expression threshold. Unfortunately, this method reduces sensitivity and hides synchronous changes between tissues that are small to moderate in global intensity. A low degree of convergence was observed among dysregulated genes in the central and peripheral mice tissues that we explored. Nevertheless, one would expect from ideal peripheral biomarkers that they reflect systemic modifications. RRHO is a recent technique developed to analyze rank changes using a threshold-free algorithm and to highlight genes presenting similar expression variations between several tissues (Plaisier et al., 2010; Bagot et al., 2016). We applied the RRHO technique on our data to study significant overlap between blood and brain gene expression signatures. Under conditions of stress (i.e., S-C vs. NS-C), we identified a robust overlap between ACC and DG (max −log10(P-value) = 957) in genes upregulated in both brain regions (Figure 5A). On the other hand, overlap between the results from blood and DG or ACC appeared to be weaker (max −log10(P-value) = 149 and 47, respectively). In parallel, in mice treated with fluoxetine (i.e., S-FLX-R vs. S-C), a more modest overlap was obtained between ACC and DG (max −log10(P-value) = 239) in genes commonly upregulated or downregulated in both of the brain regions, and no overlap was detected between blood and brain tissues (max −log10(P-value) = 2 and 7, respectively; Figure 5B). So, in our hands, the RRHO technique improved the ability to uncover shared gene expression variations between tissues. New lists of overlapping genes brought us access to potential biomarkers. We first selected genes with expression variations positively correlated between all three tissues, and varied in opposite ways under stress alone and after fluoxetine response. We identified 15 candidates (5 overexpressed and 10 underexpressed in S-C vs. NS-C comparison; Supplementary Table S6A). We identified *Cenpo*, *Mpp1* and *Tbc1d10c* which were genes also dysregulated in our previous human transcriptome data (Belzeaux et al., 2012; already validated from the classical threshold method selection) and identified three additional candidates: Fused in sarcoma *(Fus)*, Immunoglobulin mu binding protein 2 *(Ighmbp2)*, and Nucleotide binding protein 1 *(Nubp1)* for validation (Supplementary Table S6A, bold genes). Secondly, we extracted genes whose expression variations were negatively correlated between blood and the brain, and were different between S-C and NS-C mice groups. By analyzing gene ontology terms from the candidate gene lists reflecting blood-DG or blood-ACC inverted correlation (Supplementary Table S6B), we observed an enrichment for ribosomal components (Figures 5C,D). Of note, the threshold method and RRHO analyses brought out ribosomal and chromosomal factors as involved in systemic response to fluoxetine after chronic stress. Next, among the 19 candidates reflecting blood-DG inverted correlation (Supplementary Table S6B), we selected Ribosomal protein L35a (*Rpl35a*) for validation. Of the 106 candidates highlighting blood-ACC inverted correlation (Supplementary Table S6B), we selected Hexokinase 1 (*Hk1*) and Nascent polypeptide-associated complex alpha subunit (*Naca*) for validation. Of these six candidate genes, RT-qPCR assay on blood tissues revealed that *Ighmbp2*, *Nubp1* and *Rpl35a* were indeed dysregulated by stress and restored in normal levels in responders to fluoxetine. A trend to recovery was observed for *Fus* and *Hk1* (Figure 6), whereas the level of expression of *Naca* amplicon was too low to be analyzed (data not shown).

### Correlation between Stressed Mice and MDE Patients

**Figure 7:**
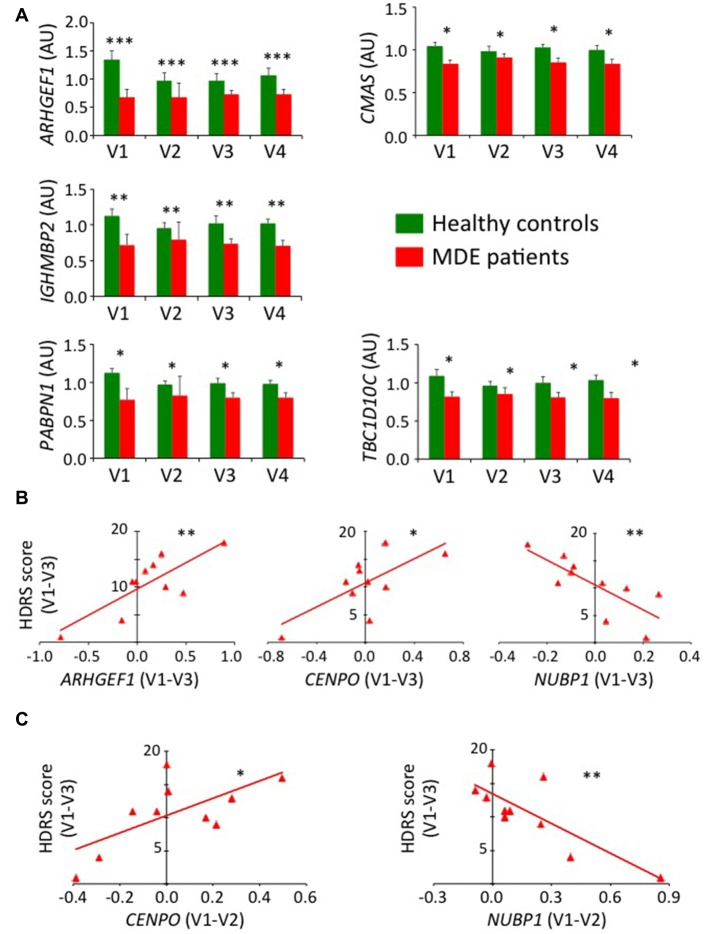
Validation of candidate gene expression in human blood. **(A)** Histograms represent mean of expression of *ARHGEF1, CMAS, IGHMBP2, PABPN1* and *TBC1D10C* on human blood. RT-qPCR data were calibrated by the mean of control samples at the four visits and normalized using *CRYL1*. Error bars denote standard error. Statistical analysis was realized using ANOVA of repeated measurements (*P < 0.05, **P < 0.01, ***P < 0.001). **(B)** Graphs represent linear regression analysis (*P < 0.05, **P < 0.01) between the variation of gene expression in major depressive episode (MDE) patients along the first 8-week of follow-up (V1–V3) and the variation of HDRS score during the same period of time for *ARHGEF1* (left panel), *CENPO* (middle panel) and *NUBP1* (right panel). **(C)** Graphs represent linear regression analysis (*P < 0.05, **P < 0.01) in MDE patients between the variation of gene expression along the first 2-week of follow-up (V1–V2) to predict the variation of HDRS score during the first 8-week of follow-up (V1–V3) for *CENPO* (left panel) and *NUBP1* (right panel).

Identification of biomarkers in a depression mice model and the translation of these biomarkers to human clinical practice constitute an important goal in biological psychiatry. Consequently, we evaluated if our previous results were transposable to human subjects. We already knew, according to our selection procedure, that some of our candidate genes were dysregulated in human MDE patients compared to controls in a naturalistic cohort. In fact, among the 13 candidate genes tested for validation in mice tissues, 11 were dysregulated in human blood samples of MDE patients (Supplementary Table S7). To extend the similarity between the murine protocol and human disease, we chose to test all validated candidate genes in mice blood samples in samples of severe MDE patients free of antidepressant treatment at inclusion (*n* = 10), as well as on age- and sex-matched healthy controls (*n* = 10). In addition to assess the possibility of either a predictive or a monitoring value of candidate gene expression, we assayed RT-qPCR expression along a 30-week monitoring corresponding to four visits: V1 (baseline), V2 (2 weeks later), V3 (8 weeks later) and V4 (30 weeks later). Among the 13 tested candidate genes (Figure 7A and Supplementary Figure S5), six genes may potentially be trait biomarkers for MDE, as they exhibited a trend or a significant variation that distinguishes the depressive and non-depressive individuals after ANOVA of repeated measurements (*ARHGEF1*, *F* = 16.2, *P* = 0.00097, *η*^2^ = 0.504; *CMAS*, *F* = 6.60, *P* = 0.021, *η*^2^ = 0.292; *IGHMBP2*, *F* = 13.2, *P* = 0.0022, *η*^2^ = 0.452; *MPP1*, *F* = 3.35, *P* = 0.082, *η*^2^ = 0.177; *PABPN1*, *F* = 7.63, *P* = 0.014, *η*^2^ = 0.323 and *TBC1D10C*, *F* = 4.86*, P* = 0.043, *η*^2^ = 0.233), while no effect of the visit alone or its interaction with the group was observed. Also, a trend for significance was obtained for *ACSL1* (Supplementary Figure S5) when considering both group distinction (*F* = 3.20, *P* = 0.092, *η*^2^ = 0.167) as well as interaction between the group and the visit parameters (*F* = 2.35, *P* = 0.084, *η*^2^ = 0.128).

To go further into the applicability of the potential biomarkers identified with the UCMS mouse model, we surmised the existence of a direct correlation between the variation of expression level of candidate genes and the evolution of the disease severity in MDE patients assessed with the HDRS score. Thus, we used with linear regression to study the candidate gene expression variation from baseline to the 8-week visit with the HDRS score variation during the same period. As shown on Figure 7B, strong associations were found for *ARHGEF1* (*b* = 9.48; 95% CI [3.69, 15.27]; *P* = 0.005), *CENPO* (*b* = 10.85; 95% CI [1.75, 19.95]; *P* = 0.025) and *NUBP1* (*b* = −22.78; 95% CI [−38.30, −7.25]; *P* = 0.010), whereas a trend was obtained for *FUS* (*b* = −12.34; 95% CI [−24.89, 0.22]; *P* = 0.053; Supplementary Figure S6). We then wondered whether candidate gene expression during the first 2 weeks of follow-up could be predictive of clinical score outcome at 8 weeks. Accordingly, variation of *CENPO* (*b* = 13.08; 95% CI [1.56, 24.60]; *P* = 0.031) and *NUBP1* (*b* = −14.79; 95% CI [−23.28, −5.77]; *P* = 0.005) expression supported their potential as treatment biomarkers (Figure 7C).

## Discussion

In the present work, we used a rodent model of major depression to show that whole blood transcriptional signatures provide a mirror to behavioral symptoms, including the response to antidepressant therapeutics. The venous signatures appeared superior to those of specialized brain regions, DG and ACC, known to be involved in the control of emotion and affective behaviors. Such an important observation reminds us that the correct and rapid recognition/treatment of a disabling condition such as MDD is an absolute imperative for the whole community (Girardi et al., 2009). Because obtaining of blood samples is easy and rapid, we think that such procedure should be incorporated as a routine for improving the interpretation of future clinical trials as well as for daily practice and decision of the psychiatrist to adapt antidepressant treatment and progressively reach a personalized medicine.

Only a few previous studies compared gene expression patterns between peripheral and central tissues in rodent models of psychiatric disorders. Daskalakis et al. (2014) demonstrated convergent signaling pathways between the blood and the brain (amygdala and hippocampus) associated with trauma-related individual differences in a rat model of posttraumatic stress disorder. Apart from a recent study comparing gene expression alterations in the medial prefrontal cortex and blood cells of ovariectomized mice subjected to chronic mild stress (Miyata et al., 2016b), to our knowledge, our study is the first to report central and peripheral transcriptional signatures associated with response to antidepressant treatment in an animal model of major depression.

Recently, translational studies have been conducted to trigger the discovery of protein-coding transcriptional markers that would play a role in the balance between susceptibility and resilience to acute and chronic signals involved in depressive symptoms (Pajer et al., 2012; Malki et al., 2015; Bagot et al., 2016; Miyata et al., 2016a,b). In a series of experiments, Redei et al. (2014) used genetic models of depression and chronic stress on different strains of rats to separately profile transcriptional signatures of depression in the brain and the blood, and test whether a subset of transcripts that differentiated depressed-like rats from non-depressed-like rats would also differentiate human patients with early-onset MDD from those without any disorder. The same gene candidates were tested for their capacity to follow and predict response to a cognitive behavioral therapy (Andrus et al., 2012; Pajer et al., 2012; Redei et al., 2014; Redei and Mehta, 2015; Mehta-Raghavan et al., 2016). Although characteristics of the animal model we used and that of our human validation cohort were different, it turned out that some variations in transcriptional signatures were shared between the different animal models and the human cohorts. For example, *CMAS*, encoding cytidine monophosphate N-acetylneuraminic acid synthetase, regulates brain sialylation, which is important for the development of brain structure and function (Yoo et al., 2015). *CMAS* was among the short list of blood-based biomarkers from the Redei et al. (2014) studies, as well as among the candidate gene list from a transcriptome analysis on a naturalistic cohort of MDE patients responding to antidepressant treatment (Belzeaux et al., 2012). *CMAS* is also part of the most dysregulated transcripts in the UCMS procedure between stressed mice responding to fluoxetine and stressed mice that were either untreated or not responding to fluoxetine (Supplementary Table S5). Our qPCR results on a validation cohort of MDE patients untreated at baseline, and demonstrating a constant different pattern of expression between patients and healthy controls along remission and antidepressant treatment, could provide to *CMAS* the status of a trait marker for MDD (Figure 7).

To select gene candidates after microarray hybridization, we relied on both the classical method of arbitrary thresholds of differential expression between the groups of mice but also on a more innovative RRHO procedure that allows us to identify patterns of overlap between gene expression profiles among two tissues (Plaisier et al., 2010). Such method has recently been used to highlight adaptive gene networks in brain involved in stress and depression susceptibility (Bagot et al., 2016; Descalzi et al., 2017). Importantly, among the top co-dysregulated processes involved in resiliency/susceptibility to depression was the ribosome. It has also been observed from work on postmortem brains of individuals with major psychiatric disorders that protein synthesis, essentially through dysregulation of ribosomal genes and messenger RNA processing, was a pathway of central importance to psychiatric health (Darby et al., 2016). Our own results are convergent with these findings, with the addition of ubiquitin-mediated proteolysis (Figures 3C, 5C), that has also been previously regarded as crucial in the antidepressant treatment response in both mice and humans (Park et al., 2017). Thus, our candidate gene list contained two players involved in the translational machinery: the immunoglobulin mu-binding protein 2 (*IGHMBP2*), encoding a DNA/RNA helicase (De Planell-Saguer et al., 2009), and *RPL35A*, encoding a component of the large subunit of the ribosome. *RPL35A* was found to be among the most ubiquitinated proteins during senescence (Bengsch et al., 2015). In addition, we noticed that among our lists of gene transcripts dysregulated by chronic stress in mice (*P* = 0.01, data not shown), and also differentially expressed between MDE patients responding to treatment and healthy controls (*P* = 3.9E-08), the *RPL24* gene was listed among 6 genes predicting remission prior to antidepressant treatment for two North American cohorts of MDD patients treated with citalopram (Guilloux et al., 2015). Among genes involved in mRNA processing, our list of biomarker candidates also included *FUS*, encoding a multifunctional protein component of the heterogeneous nuclear ribonucleoprotein complex involved in pre-mRNA splicing and the export of fully processed mRNA to the cytoplasm. We also included *PABPN1*, encoding an abundant nuclear protein that binds to nascent poly(A) tail to control its size.

Of the 13 identified genes that we tested for biomarker validation in human MDE patients, one of the genes *HK1* has been previously linked to mood and psychotic disorders. *HK1* encodes hexokinase 1, an initial and rate-limiting enzyme of glycolysis (Regenold et al., 2012). It has been known that genes associated with energy production are altered in postmortem brains as well as in peripheral tissues of MDD patients (Sibille et al., 2004; Klempan et al., 2009; Tobe, 2013; Garbett et al., 2015), and that molecular entities that are part of glycolysis pathway and the mitochondria in general serve as biomarkers and potential therapeutic targets for diagnosis and treatment of depression (Gormanns et al., 2011). Therefore, it was not surprising that the biological processes of the mitochondria are shared by the blood and the brain and are differentially regulated by chronic stress and fluoxetine treatment (Figure 3C). Another gene candidate that we tested, *ACSL1* (Acyl-CoA Synthetase Long-Chain Family Member 1), encodes an isozyme of the long-chain fatty-acid-coenzyme A ligase family and playing a key role in lipid biosynthesis and fatty acid catabolism. Cardiovascular disease is a major comorbidity of major depression and most MDD patients have unfavorable lipid profiles, likely through the effects of stress exposure (Chuang et al., 2010). We also tested the expression of *TBC1D10C*, which encodes a protein demonstrated to bind and inhibit Ras and Calcineurin (involved in the mechanism of action of antidepressants (Crozatier et al., 2007; Seimandi et al., 2013)). Such binding inhibits stress signaling but also plays a role in enhancing exercise capacity and survival (Volland et al., 2016). Furthermore, the possible role of cytoskeleton dysfunction in the pathogenesis of major depression has been already reviewed (Wong et al., 2013). We were interested in *MPP1*, which encodes a protein that helps microtubule polymerization during cell division and is also involved in remodeling the cytoskeletons of neurons (McNeely et al., 2017). *RALGPS1* and *ARHGEF1* are two gene candidates with yet unknown function but were also found to be dysregulated in both blood and brain tissues (Figures 3A,B). They belong to the family of guanine nucleotide exchange factors that participate in a broad range of cellular processes such as proliferation, differentiation and migration. Perhaps the most interesting gene candidates from our survey are the putative prediction biomarker *NUBP1*, which encodes an ATP binding protein that regulates centrosome dynamics, microtubule organization and actin cytoskeleton (Schnatwinkel and Niswander, 2012; Ioannou et al., 2013), as well as the putative state marker *CENPO*, within which an SNP loci has been associated with cognitive performance at the genome-wide significance level (Trampush et al., 2017). *CENPO* encodes a component of the interphase centromere complex, and is therefore involved in the cell cycle, one of the most dysregulated cellular processes we found for UCMS paradigm (Figure 3C). Cell cycle regulation is one of the mechanisms underlying the response to anti- and pro-neurogenic stimuli in major depression (Klempan et al., 2009; Patricio et al., 2013).

Using an animal model provides a more homogeneous genetic background to decipher pathophysiological mechanisms involved in disease evolution and its treatment, compared to using human subjects. Nevertheless, we could observe that variabilities in behavior also exist among laboratory mice, something that has not been extensively examined and tend to be minimized. An explanation for the discrepancies in behaviors within a very similar genetic background may be related to the epigenetic mechanisms by which chromatin structure and nucleosome positions are specified and maintained, as recently shown by Sun et al. (2013, 2015) for the development of susceptibility to depression and regulation of stress-related behaviors. Accordingly, Lepack et al. (2016) have recently reported a role for histone variants H3.3 dynamics in the nucleus accumbens in the regulation of aberrant social stress-mediated gene expression and the precipitation of depressive-like behaviors in mice. Indeed, our microarray data are concordant with their results, as we detected in the blood of UCMS mice a significant decrease of *H3F3a*, which is restored after fluoxetine treatment, concomitant with an opposite pattern of expression for *H3F3b* (data not shown). Moreover, we designed our study with the assumption that variations of gene expression in specific areas of the nervous system, as well as within specific cell types such as neuronal or glial cells, are mirrored by variations in the same genes in white blood cells. We do not know how cells with very different functions could have similarities in gene expression patterns and whether such patterns of expression would have any specific function for blood cells, or whether just represent a marker of a specific state of the body. One epigenetic mechanism that could be involved in conveying information from the central nervous system to the periphery involves the production, dissemination and engulfment of microRNAs that have the ability to modulate expression of hundreds of protein-encoding genes over long distances, similar to the mechanism of action of hormones (Cortez et al., 2011). Recent reports have described the role of these small RNAs in stress response, resiliency, and psychiatric disorders (Issler and Chen, 2015). It will be important in the future to establish, in the context of major depression pathophysiology, how small RNAs functionally link specific cell-types in the central nervous system, to blood cells in the periphery of the body.

We learned from the epigenomics field that DNA and RNA modifications occur in relation to environmental interactions in a dynamic fashion (Nagy and Turecki, 2012). Therefore, the above observations also emphasize the need to evaluate the pattern of RNA expression and modification at different time points. Such a task is absolutely impossible when relying on postmortem samples. It is therefore important to obtain and analyze blood samples collected from MDE patients and matching healthy control subjects over extended periods of time.

Our study has several limitations. The majority of rodent studies involving behavioral analyses used male rodents, despite a higher prevalence of MDD in women. Future investigations will require the inclusion of both sexes in animal testing (Hodes et al., 2017), but it is interesting to note that in a mouse model of depression during menopause, Miyata et al. (2016b) also found that the gene expression alterations induced by ovariectomy were mainly associated with ribosomal and mitochondrial functions in both the medial prefrontal cortex and the blood, strengthening the results we discussed above. We also only addressed the response to antidepressant treatment with a single drug, fluoxetine, while other drugs with different selectivity for neurotransmitters other than serotonin are frequently used in the clinic (Bagot et al., 2017). Other modalities such as repeated transcranial magnetic stimulation have been increasingly proposed, especially to patients resistant to first and second-line antidepressant drug treatments. Further investigations with various drugs and/or tools are thus warranted. We also classified animals as a responder or nonresponder according to a limited set of behavioral testing. Although it is not a trivial task to correlate the human antidepressant responses to that of rodents, additional behavioral testing could assess various dimensions of disease. Moreover, the stratification of stressed animals into responders and nonresponders yielded one group with only three animals. We thus cannot exclude that part of our analysis is underpowered and would require replication with larger groups of animals. In addition, it would be useful to retrieve blood profiling at the peak of the depressive symptoms and not just after a fixed period of time as there is an inter-individual variability in stress susceptibility. Finally, it will be necessary to repeat these experiments in several human cohorts, especially because large cohorts of patients are required for statistical power. It will also be important to assess the robustness of biomarker candidates by controlling for age, sex and the history of trauma, including the childhood trauma and the stress during the prior year.

In conclusion, our study confirms the power of using blood samples collected during antidepressant treatment to study a psychiatric disorder and validate the applicability of animal models to human disease in identifying targets and pathways for the design of biomarkers and novel therapeutics. Future studies should focus on biological pathways related to dynamics of genetic and epigenetic activation and repression, as targets for possible biomarkers and novel therapeutics.

## Author Contributions

CB and ECI designed the study. CB, RB and ECI obtained funding for the study. A-MLG and SL conducted the UCMS procedures. MH and ECI extracted total RNAs from mice tissues. M-NL, WE-H and RB recruited the human subjects. ECI extracted total RNA from the human cohort. MH and NF-N performed microarray procedures. AB conducted all bioinformatic analysis related to microarray raw data and data uploading on a public database. MH performed the RT-qPCR experiments. J-LC and RB collected the human clinical data. J-LC performed the statistical analyses. MH and ECI analyzed the results and wrote the manuscript. All authors approved the final version of the manuscript.

## Conflict of Interest Statement

The authors declare that the research was conducted in the absence of any commercial or financial relationships that could be construed as a potential conflict of interest.

## Footnotes

^1^http://www.ncbi.nlm.nih.gov/geo/

^2^https://david.ncifcrf.gov/
