# Supplementary material for: Translational Identification of Transcriptional Signatures of Major Depression and Antidepressant Response
Source: Front Mol Neurosci. 2017 Aug 8;10:248. doi: 10.3389/fnmol.2017.00248 (PMC5550836; doi:10.3389/fnmol.2017.00248)
Supplement: Supplementary file 1 [file Data_Sheet_1.docx]

**-- SUPPLEMENTARY INFORMATION--**

**Translational identification of transcriptional signatures of major depression and antidepressant response**

**Mylène Hervé^1,2^, Aurélie Bergon^3^, Anne-Marie Le Guisquet^4^, Samuel Leman^4^, Julia-Lou Consoloni^1,2,5^, Nicolas Fernandez-Nunez^3^, Marie-Noëlle Lefebvre^6^, Wissam El-Hage^4,7^, Raoul Belzeaux^1,2,5,8^, Catherine Belzung^4^, and El Chérif Ibrahim**^1,^**^2,9,^***

^1^Aix Marseille Univ, CNRS, CRN2M UMR 7286, Marseille, France

^2^FondaMental, Fondation de Recherche et de Soins en Santé Mentale, Créteil, France

^3^Aix Marseille Univ, INSERM, TAGC UMR_S 1090, Marseille, France

^4^Inserm U930 Eq 4, UFR Sciences et Techniques, Université François Rabelais, Tours, France

^5^AP-HM, Hôpital Sainte Marguerite, Pôle de Psychiatrie Universitaire Solaris, Marseille, France

^6^CIC-CPCET, AP-HM, Hôpital La Timone, Marseille, France

^7^CHRU de Tours, Clinique Psychiatrique Universitaire, Tours, France

^8^McGill Group for Suicide Studies, Douglas Mental Health University Institute, Department of Psychiatry, McGill University, Montreal, Quebec, Canada

^9^Aix Marseille Univ, CNRS, INT, Inst Neurosci Timone UMR 7289, Marseille, France

*** Corresponding author at:**

Aix Marseille Université, CNRS, Institut de Neurosciences de la Timone (INT) UMR 7289, 27 Bd Jean Moulin, 13385 Marseille Cedex 5, France.

Tel: +33 (0)4 91 32 40 73 Email address: [el-cherif.ibrahim@univ-amu.fr](mailto:el-cherif.ibrahim@univ-amu.fr) (E. C. Ibrahim)

**CONTENTS Page**

1 SUPPLEMENTARY MATERIALS AND METHODS 2

1.1 Animals 2

1.2 UCMS 2

1.3 Mice behavior 3

1.4 Microarray assay 4

1.5 Human cohort 5

2 SUPPLEMENTARY TABLES 7

3 SUPPLEMENTARY FIGURES 15

4 REFERENCES 21

# SUPPLEMENTARY MATERIALS AND METHODS

## Animals

Thirty-two 8-week-old male BALB/c were spread over four groups for the study under a 12/12 light/dark cycle (lights on at 9 PM), 21±2°C, food and water *ad libitum*. The first group (S-C, *Stressed-Control,* n=8) included mice subjected to UCMS procedure for 8 weeks (Nollet *et al*, 2013). The second group (NS-C, *No Stressed-Control*, n=8) comprised mice kept in standard housing conditions as controls for 8 weeks. In the third group (S-FLX, *Stressed-Fluoxetine*, n=8), mice were subjected to UCMS procedure during 8 weeks and treated in parallel by fluoxetine for the last 6 weeks. The fourth group (NS-FLX, *No Stressed-Fluoxetine*, n=8) corresponded to unstressed mice but treated with fluoxetine during the last 6 weeks. Fluoxetine was placed in drinking water at 120 mg/L and each mouse consumed 10-20 mg/kg/day of antidepressant treatment depending on water consumption. A general diagram of the experimental protocol is presented in Figure S1. At the end of the protocol, 0.5 mL of blood was collected from the submandibular vein and was stabilized with 1.3 mL RNAlater® solution (Ambion, Austin, TX). Mice were euthanized by CO_2_ inhalation. Brains were rapidly extracted and microdissected to recover ACC and DG samples.

All experiments on mice were carried out according to policies on the care and use of laboratory animals of European Community legislation 2010/63/EU. The local Ethics Committee (Comité d’Ethique en Expérimentation Animale de Val de Loire (CEEAVdL) number 19) approved the protocols used in this study (protocol number 2011-06-10).

## UCMS

Mice from the NS-C and NS-FLX groups were housed in groups of 4 in standard cages (21 x 38 cm), the UCMS-exposed mice have been isolated in individual home cage (8.5x22 cm) with no physical contact with the other mice. The stressors used were varied and applied in a different sequence each week in order to avoid any habituation. Stressors consisted in housing on damp sawdust (about 200 mL of water for 100 g of sawdust), sawdust changing (replacement of the soiled sawdust by an equivalent volume of new sawdust), placement in an empty cage (usually the home cage of the subject, but with no sawdust), placement in an empty cage with water (the mouse is placed in its empty cage, whose bottom has been filled up with 1 cm high water at 21°C), switching cages (also sometimes termed as social stress: the mouse from a cage A is placed in the soiled cage from mouse B, mouse B itself being absent in order to avoid aggressive interactions), cage tilting (45º), predator sounds, introduction of rat or cats faeces as well as fur in the mouse home cage, inversion of the light/dark cycle, lights on for a short time during the dark phase or light off during the light phase, confinement in small tubes (diameter: 4 cm; length: 5 cm).

## Mice behavior

Weight and coat state were measured weekly, as markers of UCMS-induced depressive-like behavior, except for the last week before sacrifice where coat state has been recorded twice separated by a 3-day interval on 7 different parts of the body: head, neck, dorsal coat (back), ventral coat (abdomen), tail, forepaws and hindpaws. In each body zone, the score is 0 if in a good state (the fur is smooth and shiny, with no tousled, spiky patches), 1 if in a bad state (fluffy on most of the body with slight staining of the fur), 0.5 if intermediate between the 0 and 1 (slightly "fluffy" with some spiky patches). At the end of the eighth week, a complementary test of nest building was realized just before sacrifice. For such test, mice are isolated in their home cages. This test requires to introduce pieces of cotton in the cage of the tested animal. This is done at the beginning of the activity period of the mice. The quality of the nest is scored 5 h, and 24 h later according to the scale proposed by Deacon (Deacon, 2006). To assign stress susceptibility/resiliency and then extrapolate antidepressant response/nonresponse to each mouse, cuttoffs were defined according to the distribution of the sum of both coat state measurements in NS-C and NS-FLX vs. S-C groups. Then, S-FLX mice were further divided into responders, S-FLX-R (sum of coat scores ≤ 2), and nonresponders, S-FLX-NR (sum of coat scores > 2).

## Microarray assay

Sample amplification, labeling and hybridization followed the one-color microarray-based gene expression analysis (Low Input Quick Amp Labeling) recommended by Agilent Technologies. In brief, 100 ng of each total RNA sample was reverse transcribed into cDNA using oligo dT-T7 promoter primer, then labeled cRNA was subsequently prepared from the cDNA. The reaction was performed in a solution containing dNTP mix, cyanine 3-dCTP, and T7 RNA polymerase, and incubated at 40°C for 2 h. cRNA were purified using RNeasy Mini Kit (QIAGEN, Valencia, CA) according to manufacturer’s recommendations. Dye incorporation and cRNA yield were checked with the NanoDrop ND-1000 Spectrophotometer. 600 ng of cyanine 3-labeled cRNA (specific activity > 6 pmol Cy3/μg cRNA) was fragmented at 60°C for 30 min before hybridization, for 17 h at 65°C in a rotating Agilent hybridization oven, onto Agilent whole mouse genome oligo microarrays containing 62 976 different oligonucleotide probes (55 821 without the internal quality control probes) (SurePrint G3 Mouse Gene Expression 8x60K Microarray v1, Agilent Technologies, Santa Clara, CA) and covering 39 430 Entrez Gene RNAs and 16 251 lincRNAs. Microarrays were then washed according to manufacturer’s instructions. Slides were scanned immediately after washing on the Agilent DNA microarray scanner (G2505C) using one color scan setting for 8x60K array slides. The scanned images were analyzed with Agilent feature extraction software 10.5.1.1 using default parameters (protocol GE1_105_Dec08 and Grid 028004_D_F_20110722) to obtain background subtracted and spatially detrended processed signal intensities. Blood and brain data were independently normalized by quantile normalization using limma R/bioconductor package (v.2.16.4). Only 17 368 oligonucleotide probes from blood samples and 33 264 probes from brain samples with signal intensities detectable (i.e. above background according to the “gIsWellAboveBg” Agilent feature extraction value) in ≥ 75 % of samples from one group of mice were subsequently analyzed. All the procedures from control quality steps to normalized expression matrix data export, including normalization have been performed under R language with Limma R/Bioconductor library. The microarray data are available from the gene expression omnibus (GEO, http://www.ncbi.nlm.nih.gov/geo/) under the series accession number GSE84185.

## Human cohort

Ten MDE patients (enrolled in the same city, Tours) were extracted from a larger French cohort (registered in ClinicalTrials.gov with ID: NCT02209142), and corresponded to all patients free of antidepressant at baseline, and were matched for age and sex with ten healthy controls from the same cohort. In that cohort, recruitment was naturalistic and longitudinal with clinical assessments of both patients and controls at baseline (week 0), and at 2, 8 and 30 weeks after inclusion (Table S1). All patients met the Diagnostic and Statistical Manual of mental disorders, fourth edition, Text Revision (DSM-IV-TR) criteria for major depressive episode (MDE) (American Psychiatric Association, 2000), presenting at least severe MDE (17-item Hamilton Depression Rating Scale, HDRS, score ≥ 20) (American Psychiatric Association, 2008). At the end of the first 8-week clinical follow-up, patients were classified as responders and nonresponders according to the consensual definition of clinical response corresponding to a minimal reduction of 50 % of the HDRS score from the initial evaluation. The ten healthy controls (enrolled in the same city, Marseille) were evaluated to exclude any psychiatric disorder history using the French version of standardized interview validated for health control subjects (SCID-NP) (First *et al*, 2002). All controls were also free of any medical condition and presented normal clinical and standard biological evaluation. Venous blood (8-9 mL) was drawn from fasting MDE patients and healthy controls in EDTA tubes between 7:00 and 9:00 a.m and processed within 40 min at inclusion (V1), 2 (V2), 8 (V3) and 30 (V4) weeks after inclusion. Blood was passed through a LeukoLOCK^TM^ filter (Life Technologies, Ambion, Austin, TX), which captures the total leukocyte population while eliminating red blood cells, platelets, and plasma. After rinsing with phosphate-buffered saline, the filter was flushed with RNA*later*® solution to stabilize the RNA in the captured leukocytes. The filter was then stored at –80°C before processing.

All experiments on human subjects were conducted in accordance to the latest version of the Declaration of Helsinki. The project was approved by the local ethics committee (CPP Sud Méditerranée II, Marseille, France, study registered under number 2011-A00661-40) and written informed consent was obtained after a complete description of the study to the subjects.

# SUPPLEMENTARY TABLES

**Table S1: MDE and paired control cohorts.**

| Pairs | MDE group | | | | | | | | | | | | | | Control group | | |
| --- | --- | --- | --- | --- | --- | --- | --- | --- | --- | --- | --- | --- | --- | --- | --- | --- | --- |
|  |  |  | |  | Baseline | | 2 weeks | | 8 weeks | | 30 weeks | | |  | | | |
|  | Sex | Age at first visit | Episode number | | H-17 | Treatment | H-17 | Medication | H-17 | Medication | | H-17 | Medication | |  | Sex | Age at first visit |
| 1 | M | 74 | 1 | | 24 | N | 13 | SSRI, Bzd | 13 | SSRI, Bzd | | 9 | SSRI, Bzd, TMS | |  | M | 70 |
| 2 | F | 41 | 1 | | 20 | N | 11 | Psy | 6 | N, Psy | | 6 | N, Psy | |  | F | 40 |
| 3 | F | 25 | 5 | | 23 | N | 19 | N | 12 | SSRI, Mel | | 9 | SSRI, Mel | |  | F | 25 |
| 4 | F | 22 | 1 | | 26 | N | 15 | SSRI | 13 | SSRI | | 3 | SSRI | |  | F | 23 |
| 5 | F | 26 | 1 | | 26 | N | 29 | SSRI | 16 | SSRI | | 7 | SSRI, SNRI | |  | F | 29 |
| 6 | F | 43 | 3 | | 22 | N | 13 | SSRI | 6 | Ago | | 2 | N | |  | F | 45 |
| 7 | F | 51 | 1 | | 22 | N | 15 | Psy | 21 | N | | 15 | SSRI | |  | F | 52 |
| 8 | F | 21 | 2 | | 20 | N | 15 | Ata | 16 | N, Psy | | 17 | N | |  | F | 21 |
| 9 | M | 20 | 2 | | 27 | N | 25 | SSRI, Mel | 18 | SSRI, Bzd | | 5 | SSRI, Bzd | |  | M | 24 |
| 10 | F | 24 | 1 | | 28 | N | 19 | SSRI | 10 | SSRI, Mel | | 1 | SSRI | |  | F | 24 |
| **Mean** | **8F/2M** | **34.7** | **1.8** | | **23.8** |  | **17.4** |  | **13.1** |  | | **7.40** |  | |  | **8F/2M** | **35.3** |
| **s.d.** |  | **17.5** | **1.3** | | **2.86** |  | **5.72** |  | **4.89** |  | | **5.30** |  | |  |  | **16.2** |

Abbreviations: Ago, agomelatine; Ata, atarax ; Bzd, benzodiazepine ; H-17, HDRS-17; Mel, melatonin ; N, none ; Psy, psychotherapy ; SNRI, serotonin-norepinephrine reuptake inhibitor; SSRI, selective serotonin reuptake inhibitor; TMS, transcranial magnetic stimulation.

**Table S2: References of primer/TaqMan probe assays and mean of Ct.**

| **Mouse** | | | | |  | **Human** | | |
| --- | --- | --- | --- | --- | --- | --- | --- | --- |
| **Gene** | **Primer reference** | **Blood** | **ACC** | **GD** |  | **Gene** | **Primer reference** | **Blood** |
| *Acsl1* | Mm00484217_m1 | 24.75 | 25.33 | 24.99 |  | *ACSL1* | Hs00960561_m1 | 22.39 |
| *Arhgef1* | Mm00476230_m1 | 25.48 | 25.87 | ND |  | *ARHGEF1* | Hs00180327_m1 | 22.64 |
| *Cenpo* | Mm00506265_m1 | 31.91 | ND | 29.78 |  | *CENPO* | Hs00256990_m1 | 27.07 |
| *Cmas* | Mm00515534_m1 | 25.02 | ND | ND |  | *CMAS* | Hs00218814_m1 | 26.10 |
|  |  |  |  |  |  | *CRYL1 ** | Hs00211084_m1 | 26.47 |
| *Fus* | Mm00836363_g1 | 28.90 | 24.14 | 24.07 |  | *FUS* | Hs01100224_m1 | 23.58 |
| *Hk1* | Mm00439344_m1 | 26.25 | 21.55 | 21.42 |  | *HK1* | Hs00175976_m1 | 24.46 |
| *Ighmbp2* | Mm00456315_m1 | 31.93 | 27.62 | 27.11 |  | *IGHMBP2* | Hs00158054_m1 | 29.35 |
| *Mpp1* | Mm00599703_m1 | 24.01 | ND | 25.61 |  | *MPP1* | Hs00609971_m1 | 23.32 |
| *Naca* | Mm00479007_m1 | > 40 | ND | ND |  |  |  |  |
| *Nubp1* | Mm00478752_m1 | 30.35 | 26.71 | 26.19 |  | *NUBP1* | Hs00159531_m1 | 27.07 |
| *Pabpn1* | Mm00479791_m1 | 28.48 | 23.86 | ND |  | *PABPN1* | Hs01091143_g1 | 25.48 |
| *Rab5a ** | Mm00727887_m1 | 28.73 | 23.31 | 23.13 |  |  |  |  |
| *Ralgps1* | Mm00613694_m1 | 31.84 | 25.48 | 24.84 |  | *RALGPS1* | Hs01115436_m1 | 30.08 |
| *Rpl35a* | Mm03992567_s1 | 32.20 | 30.87 | 30.29 |  | *RPL35A* | Hs01086499_g1 | 20.96 |
|  |  |  |  |  |  | *SV2A ** | Hs00372069_m1 | 30.17 |
| *Tbc1d10c* | Mm00724447_m1 | 26.21 | 32.15 | 31.71 |  | *TBC1D10C* | Hs00736460_m1 | 22.59 |

* Reference gene

ND, not determined

**Table S3: Reclassification of mice based on coat scores.**

| **Former groups** | **Mice** | **Coat scores** | **New groups** |
| --- | --- | --- | --- |
|  |  |  |  |
| NS-C | 39 | 0 | NS-C |
|  | 40 | 0 |  |
|  | 52 | 0 |  |
|  | 53 | 0 |  |
|  | 54 | 0 |  |
|  | 64 | 0 |  |
|  | 65 | 1 |  |
|  | 66 | 0 |  |
| NS-FLX | 46 | 0 | NS-FLX |
|  | 47 | 0 |  |
|  | 71 | 0 |  |
|  | 72 | 0 |  |
|  | 45 | 0.5 |  |
|  | 58 | 0.5 |  |
|  | 59 | 0.5 |  |
|  | 73 | 0.5 |  |
| S-C | 25 | 2 | - |
|  | 11 | 2.5 | S-C |
|  | 19 | 2.5 |  |
|  | 35 | 3 |  |
|  | 1 | 3.5 |  |
|  | 13 | 4 |  |
|  | 15 | 4.5 |  |
|  | 32 | 4.5 |  |
| S-FLX | 8 | 1 | S-FLX-R |
|  | 7 | 2 |  |
|  | 10 | 2 |  |
|  | 22 | 2 |  |
|  | 38 | 2 |  |
|  | 17 | 2.5 | S-FLX-NR |
|  | 27 | 2.5 |  |
|  | 14 | 4.5 |  |

**Table S4: Shared transcriptional signatures between mouse tissues.**

**A**

**B**

Tables indicate shared transcriptional signatures between two or three mice tissues using classical threshold method to compare either S-C vs. NS-C and S-FLX-R vs. S-C mice groups (A, *P* < 0.05), or S-C vs. NS-C groups only but with *P* < 0.01 and *FC* > 1.2 (B). Candidate genes are indicated in bold, genes shared with a previous transcriptome analysis conducted on blood samples from MDE patients and healthy control individuals (Belzeaux et al, 2012) are underlined and common genes between classical threshold analysis and RRHO method are indicated in red.

**Table S5: Blood dysregulated genes in common through threshold method and SAM analysis.**

| 1110020G09Rik | Dhrs11 | Nusap1 |
| --- | --- | --- |
| 1700037H04Rik | Dnajb2 | Pcx |
| 2010011I20Rik | Eef1d | Pnpo |
| 2700097O09Rik | Fzr1 | Ppp2r5b |
| 5730469M10Rik | Gda | Psmd4 |
| A_55_P2063146 | Glrx | Psme4 |
| A_55_P2066429 | Glrx5 | Ptp4a3 |
| Acmsd | Gm12839 | Rab3il1 |
| Acp1 | Gm15800 | Reep6 |
| **Acsl1** | Gm4055 | Slc4a1 |
| Add2 | Gm7227 | Snrnp25 |
| Ahsp | Gypa | Snx15 |
| Ank1 | Hagh | Tmod1 |
| Asb1 | Hebp1 | Trim10 |
| B230312A22Rik | Hipk1 | Tspo2 |
| Brp44 | Hmbs | Ube2b |
| Car2 | LOC100504461 | Ube2c |
| Cat | Mcart1 | Ube2f |
| Ccnl2 | Metap2 | Ubl7 |
| chr11:95644899-95658549_R | **Mpp1** | Urod |
| chr12:32781477-32808567_R | Myo1d | Vangl1 |
| **Cmas** | Nars | Wdr26 |
| Cnnm2 | Nt5c3 | Yipf4 |
| Dcaf6 |  |  |
|  |  |  |

Common dysregulated gene list in blood after comparing the effect of stress and fluoxetine treatment (*P* < 0.05) with the threshold method to the variations observed in (NS-FLX + S-FLX-R) vs. (S-C + S-FLX-NR) groups of mice with a SAM analysis (*FDR* < 1 %). Underlined genes were also dysregulated in human transcriptome (MDD patients vs. controls, *P* < 0.05) in a previous transcriptome analysis (Belzeaux et al, 2012). Bold genes have been assayed by RT-qPCR for validation on mice samples. Red gene is common to threshold and RRHO analyses.

**Table S6: Most co-dysregulated genes under stress and after fluoxetine treatment in blood and brain tissues.**

**B**

**A**

| **Most co-dysregulated genes in NS-C vs. S-C in blood (↘) and DG (↗)** |  | **Most co-dysregulated genes in NS-C vs. S-C in blood (↘) and ACC (↗)** | | |
| --- | --- | --- | --- | --- |
| A_55_P2010018 |  | 1810063B05Rik | ENSMUST00000093411 | NAP111292-1 |
| A_55_P2033075 |  | A_55_P1953136 | ENSMUST00000096279 | NAP111430-1 |
| Btbd1 |  | A_55_P1954755 | ENSMUST00000138502 | NAP112543-1 |
| Eif2ak3 |  | A_55_P1957159 | Gfm1 | NAP113790-1 |
| ENSMUST00000080603 |  | A_55_P1958102 | Gm12270 | NAP114286-1 |
| ENSMUST00000085163 |  | A_55_P1972097 | Gm13215 | NAP114346-1 |
| ENSMUST00000091506 |  | A_55_P2010018 | Gm4613 | Nck1 |
| Gm13215 |  | A_55_P2013680 | Gm4889 | Prpf38a |
| Gm15501 |  | A_55_P2022663 | Gm4997 | Ptplad1 |
| Gm4889 |  | A_55_P2033075 | Gm5321 | Rpl14 |
| NAP027211-1 |  | A_55_P2037544 | Gm5561 | Rpl26 |
| NAP101394-1 |  | A_55_P2042312 | Gm6636 | Rpl29 |
| NAP103572-1 |  | A_55_P2049289 | Gm9104 | Rpl30 |
| NAP111430-1 |  | A_55_P2052475 | **Hk1** | **Rpl35a** |
| NAP111971-1 |  | A_55_P2090953 | Ifi204 | Rpl36a |
| Polr2i |  | A_55_P2102715 | Igf2r | Rpl36al |
| **Rpl35a** |  | A_55_P2112510 | Itpr3 | Rpl37 |
| Rps28 |  | A_55_P2128224 | Klhdc5 | Rpl5 |
| Rptor |  | A_55_P2135633 | LOC100039181 | Rps11 |
|  |  | A_55_P2141654 | LOC100502877 | Rps13 |
|  |  | A_55_P2161818 | LOC636187 | Rps17 |
|  |  | A_55_P2172233 | Med31 | Rps19 |
|  |  | A_55_P2183688 | Metap1 | Rps20 |
|  |  | A_55_P2186787 | Mobkl3 | Rps27 |
|  |  | A430005L14Rik | Mrpl15 | Rps28 |
|  |  | Anapc11 | Mrpl23 | Rps3a |
|  |  | Ap1s3 | **Naca** | Rps8 |
|  |  | Btbd1 | NAP026650-1 | Rptor |
|  |  | Canx | NAP061860-1 | Sec23ip |
|  |  | Ccnl1 | NAP062640-1 | Skil |
|  |  | Cdv3 | NAP092996-001 | Sprr2a2 |
|  |  | chr14:115443612-115445950_F | NAP093766-001 | Stt3b |
|  |  | chr2:167085700-167117795_R | NAP093810-001 | Tomm20 |
|  |  | ENSMUST00000080603 | NAP096498-001 | Usp1 |
|  |  | ENSMUST00000085163 | NAP100935-001 | Zfp217 |
|  |  | ENSMUST00000091506 |  |  |

Underlined genes were also dysregulated in human transcriptome (MDD patients vs. controls, *P* < 0.05) in a previous transcriptome analysis (Belzeaux et al, 2012). Bold genes have been assayed by RT-qPCR for validation on mice samples. Red genes are common to threshold and RRHO analyses.

**Table S7: Candidate gene expression in PBMCs from a previous human cohort.**

Abbreviations: C, controls; C_inclusion_, controls at inclusion; C_8weeks_, controls at the 8-week visit FC, fold change; P, patients; P_inclusion_, patients with severe MDE at inclusion; P_8-weeks_, patients at the 8-week visit.

Genes in bold present significant P-value at either one of the comparison between patients and controls.

# SUPPLEMENTARY FIGURES

**Figure S1: Experimental procedure on mice.**

**Figure S2: Maximal coat score reflecting depressive behavior intensity.**

Dot plot graphics represent mean of maximal coat scores obtained between the third and seventh week of the UCMS protocol in the four mice groups (A) or in reclassified categories of mice according to their phenotype (B). Error bars denote standard error (*** *P* < 0.001 using non-parametric Mann-Whitney test).

**Figure S3: Expression level of analyzable probes in blood and brain samples.**

Histograms represent mean expression intensity, for each group of mice, of 17 368 analyzable probes in blood and 33 264 probes in each brain region. Error bars denote standard error.

**Figure S4: Heatmap representation of gene expression changes in brain regions.**

**B**

**A**


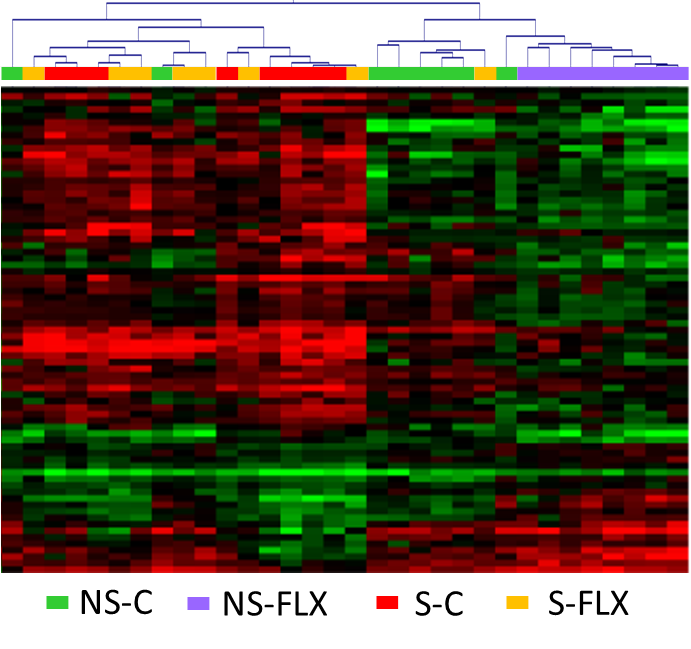


**C**

**D**


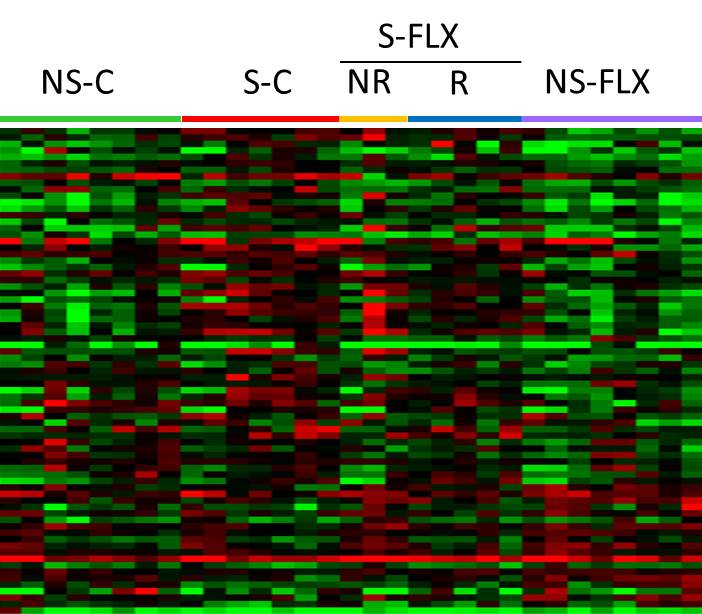

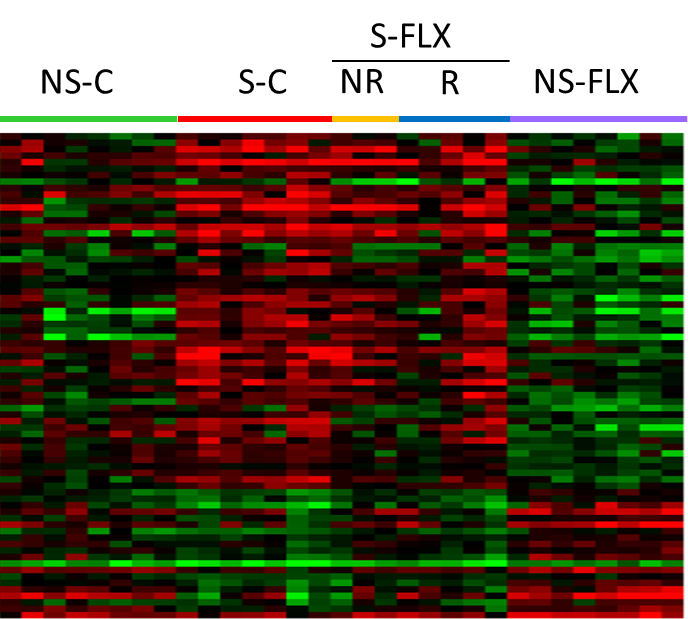


**
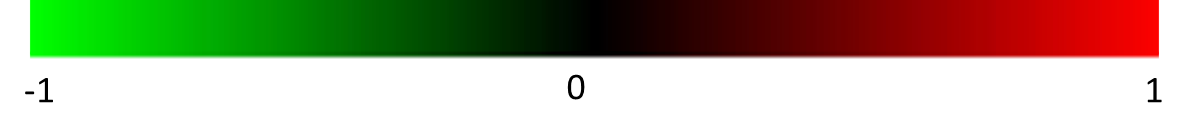
**

Heatmap of 75 gene probes expression in DG (left panel) and ACC (right panel). Overexpressed probes are in red and underexpressed probes in green. Normalized signal intensities were treated using the MeV software by applying SAM test in unsupervised mode (A-B) or supervised mode based on the five reclassified groups (C-D).

**Figure S5: Expression of *ACSL1* and *MPP1* in human blood.**

V1

V2

V3

V4

0.0

0.5

1.0

1.5

2.0

*ACSL1* (AU)

V1

V2

V3

V4

0.0

0.5

1.0

1.5

*MPP1* (AU)

Histograms represent mean of expression of *ACSL1* and *MPP1* on human blood, at the four visits for healthy controls (green) and MDE patients (red). RT-qPCR data were calibrated using mean of control samples and normalized by *CRYL1*. Error bars denote standard error.

**Figure S6: Variation of *FUS* expression in relation to clinical socre variation during 8 weeks of antidepressant treatment.**

-0.6

-0.3

0.0

0.3

0.6

20

10

*FUS* (V1-V3)

HDRS score (V1-V3)

The graph represent the linear regression analysis (*P* = 0.053) between the variation of gene expression in MDE patients along the first 8-week of follow-up (V1-V3) and the variation of HDRS score during the same period of time for *FUS.*

# REFERENCES

American Psychiatric Association A (2000). *Diagnostic and Statistical Manual of Mental Disorders, Fourth Edition, Text Revision (DSM-IV-TR)* American Psychiatric Association: Arlington, VA.

American Psychiatric Association A (2008). *Handbook of psychiatric measures*, 2d edn. American Psychiatric Association (APA): Washington, DC, 864pp.

Deacon RM (2006). Assessing nest building in mice. *Nat Protoc* **1**(3): 1117-1119.

First MB, Spitzer RL, Gibbon M, Williams JBW (2002). Structured Clinical Interview for DSM-IV-TR Axis I Disorders, Research Version, Patient Edition. (SCID-I/P). New York State Psychiatric Institute: New York.

Nollet M, Le Guisquet AM, Belzung C (2013). Models of depression: unpredictable chronic mild stress in mice. *Curr Protoc Pharmacol* **Chapter 5**: Unit 5 65.
